# Astaxanthin Supplementation Does Not Alter Training-Related Changes in Inflammatory Cytokine Profile in Arabian Racing Horses

**DOI:** 10.3390/antiox13080905

**Published:** 2024-07-26

**Authors:** Beata Giercuszkiewicz-Hecold, Marek Kulka, Michał Czopowicz, Ewa Szarska, Katarzyna Strzelec, Arkadiusz Grzeczka, Szymon Graczyk, Marta Wiśniewska, Zofia Jędrzejkowska, Aleksandra Rumińska, Krzysztof Marycz, Anna Cywińska

**Affiliations:** 1 Doctoral School, Warsaw University of Life Sciences-SGGW, Nowoursynowska 159c, 02-776 Warsaw, Poland; b.giercuszkiewicz@gmail.com; 2 Department of Pathology and Veterinary Diagnostics, Institute of Veterinary Medicine, Warsaw University of Life Sciences-SGGW, Nowoursynowska 159c, 02-776 Warsaw, Poland; marek_kulka@sggw.edu.pl; 3 Division of Veterinary Epidemiology and Economics, Institute of Veterinary Medicine, Warsaw University of Life Sciences-SGGW, Nowoursynowska 159c, 02-776 Warsaw, Poland; michal_czopowicz@sggw.edu.pl; 4 Military Institute of Hygiene and Epidemiology, Kozielska 4, 01-001 Warsaw, Poland; eszarska@gmail.com; 5 Department of Horse Breeding and Use, University of Life Sciences in Lublin, Akademicka 13, 20-950 Lublin, Poland; katarzyna.strzelec@up.lublin.pl; 6 Student of the Faculty of Biological and Veterinary Sciences, Nicolaus Copernicus University in Toruń, Lwowska 1, 87-100 Toruń, Poland; grzeczka@umk.pl (A.G.); graczyk72@gmail.com (S.G.); 310947@stud.umk.pl (M.W.);; 7 Department of Basic and Preclinical Sciences, Faculty of Biological and Veterinary Sciences, Nicolaus Copernicus University in Torun, Lwowska 1, 87-100 Toruń, Poland; 8 International Institute of Translational Medicine, Jesionowa 11, Malin, 55-114 Wisznia Mała, Poland; krzysztofmarycz@gmail.com; 9 Department of Veterinary Medicine and Epidemiology, Veterinary Institute for Regenerative Cures, School of Veterinary Medicine, University of California, Davis, CA 95516, USA

**Keywords:** astaxanthin, horse, race training, cytokines, anti-inflammatory state

## Abstract

This study aimed to evaluate the oral supplementation of astaxanthin (ATX) on inflammatory markers in 3-year-old Arabian racehorses. Despite the recognized antioxidant and anti-inflammatory properties of ATX observed in vitro in rodent models and in human athletes, the effects in equine subjects remain unknown. This study involved a controlled trial with 14 horses receiving either ATX (six horses) or a placebo (eight horses), monitored over four months of race training. Inflammatory cytokines: TNFα, IFNγ, IL-6, IL-10, and prostaglandin E (PGE), were measured monthly to assess the impact of ATX on the inflammatory response. The results indicated no significant differences in measured parameters between the ATX and the control group during the study. However, a significant time-dependent decrease in TNFα and IFNγ levels (*p* = 0.001) was observed in both groups, suggesting that regular training naturally modulates inflammatory responses. Moreover, positive correlations were noted between TNFα and IFNγ (*p* < 0.001) in the early phase of the study and between IL-6 and IL-10 (*p* = 0.008) in the later phase. Hematological parameters remained stable and within reference ranges, indicating no adverse effects of ATX supplementation. Performance metrics, including the number of races completed and wins, showed no significant differences between groups, suggesting that ATX did not enhance athletic performance under the study conditions. Overall, while ATX supplementation affected neither cytokine levels nor performance in Arabian racehorses, the natural anti-inflammatory effects of regular training were evident. Further research is needed to explore potential benefits of ATX supplementation under different conditions, such as in horses with subclinical inflammation or varying training regimens, to fully clarify its role and applications in equine sports medicine.

## 1. Introduction

Astaxanthin (ATX) is a potent carotenoid antioxidant recommended as a supplement for both humans and animals across various indications. Due to its unique chemical structure, ATX exhibits superior bioavailability and demonstrates an antioxidant potency that is tenfold greater than of β-carotene and a hundredfold greater than of α-tocopherol [1,2,3]. In the bloodstream of dogs and cats, ATX is transported by high-density lipoprotein (HDL) and is taken up by leukocytes, thereby being distributed to all subcellular structures. The biokinetic uptake of astaxanthin in dogs and cats exhibits similarities to that observed in humans [4,5], making ATX an ideal antioxidant dietary additive.

Supplementation with ATX has been extensively investigated for its multiple effects in reducing and preventing oxidative stress and tissue damage [2,6,7]. In humans, the beneficial effects of ATX include anti-inflammatory, immunostimulant [8], anti-cancer [9], and anti-diabetic properties [10]. Notably, its anti-inflammatory action has been documented not only in clinical patients but also in athletes experiencing immune perturbations and inflammatory responses due to physical exertion [11,12].

In animals, ATX-rich algae *Haematococcus pluvialis* have been recommended as feed additives to enhance the quality of animal-derived products and are approved for human consumption to a certain extent [13,14]. Additionally, ATX has shown benefits as a supplement in dogs with obesity-related inflammatory disorders [15] and horses suffering from equine metabolic syndrome [7]. Rodent models have further demonstrated that ATX may alleviate oxidative stress in mice and rats subjected to a high-fat diet [16,17,18].

Currently, astaxanthin (ATX) supplementation for horses is gaining increasing interest. It is commercially recommended for various groups of healthy horses, including those engaged in moderate to heavy work, as well as growing and elderly ones. Additionally, the use of ATX is widespread in injured, immune-compromised, and allergic horses [19,20,21], as well as horses with muscle disorders such as tying-up syndrome or polysaccharide storage myopathy (PSSM). In racehorses, antioxidants have been proposed as supplements reducing inflammation in the musculoskeletal system [22,23] and airways [24]. Due to the strong antioxidant properties of ATX and the fact that the mechanism by which it limits exercise-induced muscle damage has been recognized in mice [25], it is widely considered as a supplement for human athletes [12] and seems to be a perfect candidate also for horses. Additionally, antioxidants including ATX are not currently forbidden by anti-doping regulations.

However, the rapidly increasing popularity of ATX in horses is scarcely supported by scientific evidence. It has been shown that long-term supplementation with ATX and L-carnitine is associated with lower serum creatinine kinase (CK) activity and a tendency toward lower lactate dehydrogenase isoenzyme-5 (LDH-5) activity, suggesting these compounds may reduce the rate of exercise-induced muscle damage [22]. Current anti-inflammatory recommendations for horses are based only on in vitro studies, rodent studies, and observations in humans [26,27,28,29]. In the last decade, interest in the welfare of the exercised horse has grown considerably since the animals must face potentially stressful situations during their training and work. Hence, evaluating their stress response could be the primary approach to success in the horses’ performance and welfare, as described in common equestrian disciplines [30]. An inflammatory response triggered by exercise has been documented in both humans [31,32,33] and horses [34,35,36]. This response involves musculoskeletal microdamage and metabolic derangements related to oxidative stress. However, regular training leads to adaptations that include a reduction in inflammation, sometimes described as an “anti-inflammatory state” [37,38]. It has also been reported that this reduction in inflammatory response in young racehorses may be further enhanced by nutritional supplementation with curcumin, *Boswellia*, coenzyme Q10, L-carnitine, and D-ribose [23]. Therefore, it has been hypothesized that astaxanthin supplementation may have a similar effect.

The objective of this study was to evaluate changes in serum concentrations of inflammatory markers (cytokines and prostaglandin E, PGE) during the initial months of race training in Arabian horses. A reduction in training-related inflammatory responses in ATX-supplemented horses, expressed as either an earlier or more pronounced decrease in the concentrations of inflammatory markers was anticipated.

## 2. Materials and Methods

### 2.1. Horses and Training

This study included 14 (9 stallions and 5 mares) privately owned 3-year-old Arabian horses under training for flat races. All horses were kept in stables belonging to the Służewiec Racetrack in Warsaw and fed on a diet composed of hay, oats, and concentrate satisfying the nutritional recommendations for racing Arabians. Salt and water were available ad libitum. The horses were trained by the same trainer and were at a similar training level. They were clinically examined by a certified veterinary surgeon as well as dewormed and vaccinated according to the routine schedule, not earlier than 3 weeks before the onset of the study. At enrollment, the horses were examined for gait regularity and basic clinical measurements (rectal temperature, heart rate, respiratory rate, mucous membranes, respiratory sounds, and gut sounds) and classified as healthy and fit for race training. Before and after races, the horses underwent the routine health check provided by a certified veterinary surgeon according to the regulations applied for races. Concomitant disabilities or diseases were the criteria for exclusion from the study.

At the beginning of the study (in April), the horses were randomly (simple randomization based on a pseudorandom number generator) allocated to the ATX group (4 stallions and 2 mares) and control group (5 stallions and 3 mares). The ATX group was supplemented with ATX at a daily dose of 250 mg per horse, which corresponded to a dose of 0.52–0.58 mg/kg body weight. The control group did not receive any supplementation.

**Figure 1:**
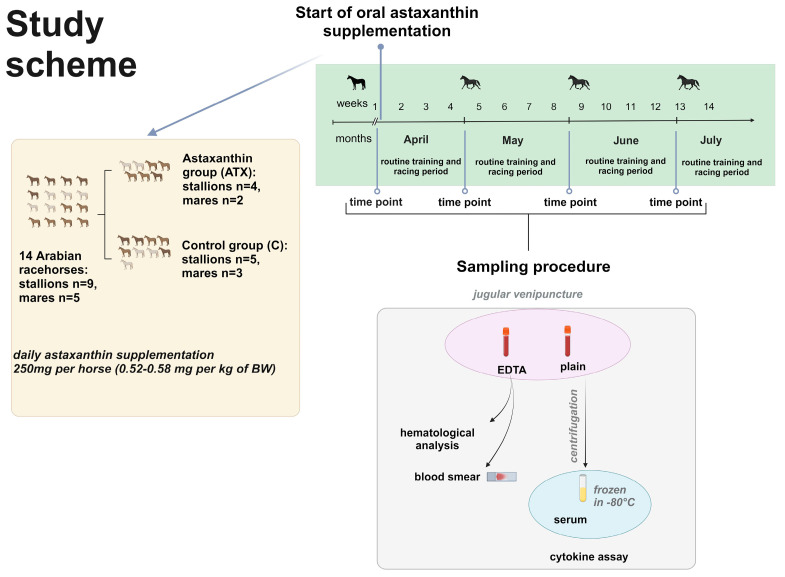
Study design. Created with BioRender.com.

Stallions and mares trained together with the same intensity at the Służewiec Racetrack in Warsaw according to the exercise schedule involving 2 intensive training sessions every week. The intensive training sessions included warm-up walking and trotting with a rider (about 15 min), followed by cantering and a fast gallop (45–50 km/h) for 800 m, and then 40 min. of exercise in a horse walker. To evaluate the long-term effect of astaxanthin supplementation (Figure 1) on the inflammatory markers, the horses were examined 4 times: in April just before they started intensive training sessions and ATX supplementation, and then monthly before the intensive training sessions—in May after 1 month of training, in June after 2 months of training, and in July after 3 months of training.

### 2.2. Blood Sampling

During the sampling procedure, the horses were handled by their regular riders to minimize stress, as recommended by the Ethical Committee guidelines. All of the procedures of blood sampling were performed as part of routine health examinations, and thus, according to the European directive EU/2010/63 [39] and Polish regulations regarding experiments on animals, there was no need for the approval of the Ethics Committee for the described procedures, which qualified as non-experimental clinical veterinary practices, and were excluded from the directive. A written consent for the use of blood for scientific analyses was obtained from the trainer.

Blood samples were collected from the jugular vein, at rest, before each training session between 6:00 and 6:30 a.m. Samples were collected into BD Vacutainer (Becton Dickinson, Reading, UK) EDTA tubes (Becton Dickinson, UK) for hematological analysis, and dry (serum) tubes (Becton Dickinson, UK) for cytokine and PGE analyses. All samples were stored at 4 °C and EDTA samples were analyzed within 6 h after collection. Blood in dry tubes was allowed to clot and centrifuged for 10 min at 1000× *g*. Then, the serum was harvested and kept at −80 °C until cytokine and PGE quantification. The following hematological parameters were measured using an automated hematology analyzer (Sysmex XN-10, Lincolnshire, IL, USA): red blood cell count (RBC), hemoglobin concentration (HGB), hematocrit (HCT), white blood cell count (WBC), neutrophil count (NEU), lymphocyte count (LYM), monocyte count (MON), eosinophil count (EOS), basophil count (BAS), and platelet count (PLT). Leukocyte differential count and blood cell morphology were assessed on high-quality peripheral blood smears stained with the May–Grünwald–Giemsa reagent using a light microscope (Primo Star, Zeiss, Jena, Germany) under 1000× magnification.

### 2.3. Cytokine and PGE Assays

The concentrations of 4 cytokines (tumor necrosis factor α—TNFα, interferon γ—IFNγ, interleukin 6—IL-6, and interleukin 10—IL-10) and one prostaglandin (PGE) were measured using immunoenzymatic commercial assays dedicated for equine species (Cloud-Clone Corp., Katy, TX, USA) according to the manufacturer’s protocols. Serum samples for PGE quantification were diluted 1:2. The absorbance was measured with a Multiscan Reader (Labsystem, Helsinki, Finland) using a Genesis V 3.00 software program. Intra- and inter-plate precision of the assays (expressed as the coefficient of variation; CV) were CV < 10% and CV < 12%, respectively. The quantification range was 15.6–1000 pg/mL for the TNFα, IFNγ, and IL-6 assays, 7.8–500 pg/mL for the IL-10 assay, and 24.7–2000 pg/mL for the PGE assay.

### 2.4. Statistical Analysis

Numerical variables (hematological measurements as well as cytokine and PGE concentrations) were examined for normality of distribution using the normal probability Q-Q plots and the Shapiro–Wilk test. As the normality assumption was violated (*p* < 0.001 for TNFα, IL-6, IL-10, PGE, and *p* = 0.01 for IFNγ), they were summarized using the median and range in tables and median, interquartile range (IQR), and individual measurements in figures. Cytokine concentrations were compared between two groups at each time point using the Mann–Whitney U test. If no significant difference was detected, the groups were merged and the change of cytokine concentrations in time was assessed using the Friedman test. If the latter proved significant, it was followed by the Wilcoxon signed-rank test to identify time points at which the cytokine concentrations significantly differed. The Mann-Whitney U test was also used to compare the numbers of races and wins between the two groups. Correlations between the cytokine and PGE concentrations were analyzed on the common group of 14 horses using the Spearman rank correlation coefficient (R_s_). All tests were two-tailed, and the significance level (α) was set at 0.05. No correction for multiple comparisons was applied to avoid increasing the type II error (i.e., to increase the power of statistical comparisons negatively affected by small group sizes). The statistical analysis was performed with TIBCO Statistica 13.3 (TIBCO Software Inc., Palo Alto, CA, USA).

## 3. Results

All horses trained regularly during the study period and did not exhibit visible muscle damage or any other pathological conditions. Most horses were presented at official races and the number of completed races as well as the number of wins did not differ significantly between the groups (*p* = 0.662 and *p* = 0.852, respectively) (Table 1). Five horses were sampled three instead four times (one month was missing) and one horse was sampled only twice due to the race plan. The horses were not sampled if they were planned to participate in a race the following day. Missing values were replaced by the arithmetic mean calculated from the measurements of all horses examined in a corresponding month.

The basic hematological results (WBC, RBC, HCT, and HGB) remained within the reference intervals [40] during the entire study (Table 2) and in the subsequent months. No abnormalities were found in the microscopic examination of the blood cells.

**Figure 2:**
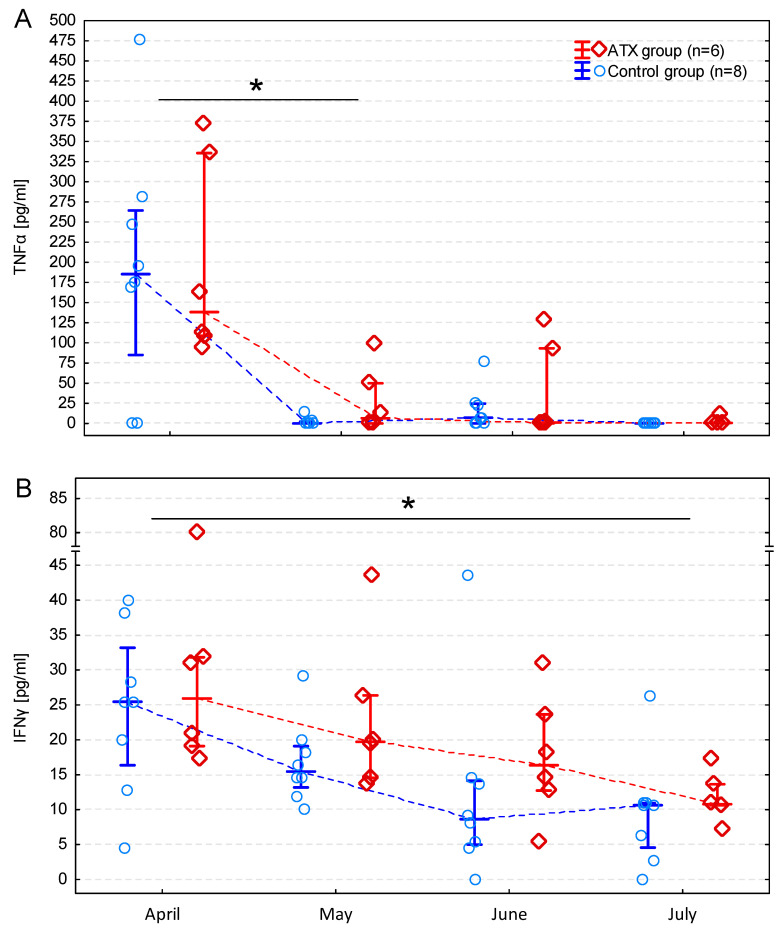
Changes in tumor necrosis factor α (TNFα) (**A**) and interferon γ (IFNγ) (**B**) concentrations in the subsequent months presented as medians, interquartile ranges, and individual measurements in the astaxanthin (ATX) and control groups. Asterisks (*) indicate significantly different time points according to the Wilcoxon signed-rank test.

The concentrations of TNFα, IFNγ, IL-6, IL-10, and PGE did not differ significantly between groups; however, the time effect was significant in terms of TNFα and IFNγ (Table 3). The concentration of TNFα was the highest in April, then decreased significantly in May (*p* = 0.009) and remained significantly lower in June (*p* = 0.015) and July (*p* = 0.007) (Figure 2A). Similarly, the concentration of IFNγ was the highest in April and then decreased gradually, reaching a significantly lower value in July (*p* = 0.006) (Figure 2B). The concentrations of IL-6 and IL-10 did not change significantly during the study period (Table 3).

**Figure 3:**
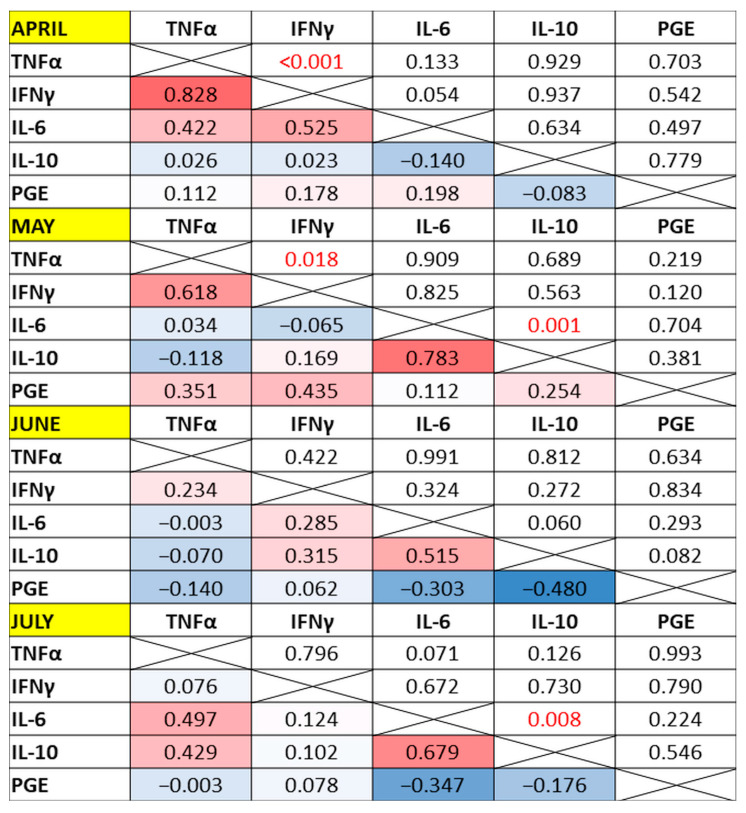
A correlation heatmap showing Spearman’s rank correlation coefficients among the five cytokines in 14 horses at the subsequent time points (cells below the diagonal) and their *p*-values (cells above the diagonal). The intensity of the color indicates the closeness of the value to 1 (red) or −1 (blue). The *p*-values in red are those considered significant at α = 0.05.

When all 14 horses were analyzed together, significant positive correlations were observed between TNFα and IFNγ concentrations in April (R_s_ = 0.83, *p* < 0.001) and May (R_s_ = 0.62, *p* = 0.018) and between IL-6 and IL-10 concentrations in May (R_s_ = 0.78, *p* = 0.001) and July (R_s_ = 0.68, *p* = 0.008) (Figure 3).

## 4. Discussion

Despite the anticipated benefits of ATX due to its strong antioxidant and anti-inflammatory properties observed in vitro, in rodent models, and in human athletes, the results of this study did not indicate a significant effect of ATX on cytokine concentrations in the blood of Arabian horses training for flat races. The concentrations of TNFα, IFNγ, IL-6, IL-10, and PGE did not differ significantly between the ATX and control groups throughout the study period.

The literature data have shown that ATX exerts an anti-inflammatory effect by suppressing the NF-κB transcription factor, leading to a decrease in the levels of proinflammatory cytokines, such as IL-1β, IL-6, and TNFα [8,27,28,41]. A recent in vitro study has confirmed that ATX affects the whole network of inflammatory factors, with a particular effect on IL-6, and also protects the cells from inflammatory damage by stimulating p53 and inhibiting transcription factor STAT3 [42]. Moreover, ATX regulates the Th1/Th2 balance leading to a decrease in Th2 cytokine levels (IL-4 and IL-5) and an increase in Th1 derived IFN-γ [8,43,44]. Additionally, ATX inhibits the production of other inflammatory mediators by suppressing the activation and/or protein degradation of inducible nitric oxide synthase (iNOS) and cyclooxygenase 2 (COX-2) [8,29]. Possibly the anti-inflammatory mechanism is interrelated with the antioxidant effect of ATX [41], which additionally encouraged its use in racing horses. The selection of inflammatory mediators measured in this study was based on the mechanisms listed above. It was expected that in supplemented horses the concentrations of the above-mentioned mediators would decrease earlier or in a more pronounced manner, as has been reported in literature in other conditions.

The anti-inflammatory effect of ATX expressed as the changes in cytokine concentrations has been evidenced in vitro and in rodent models, as well as in people with chronic inflammatory conditions. Significant decreases in serum levels of IL-1β and TNFα were observed in women with endometriosis treated with ATX for 12 weeks [45]. In diabetic patients, the serum IL-6 level decreased after 8 weeks of ATX supplementation [46]. The 14-week supplementation in the present study seems long enough to exert an apparent effect; however, no significant changes occurred. In the literature, IL-6 and TNFα have been investigated most often and their changes after ATX supplementation were the most pronounced. Interestingly, a meta-analysis of randomized controlled clinical trials confirmed the reduction of IL-6 levels upon ATX supplementation, but not the decreases in TNFα levels [47,48,49]. In non-rodent animals, the proinflammatory cytokines were studied only in vitro, in a bovine endometrial epithelial cell line, and ATX reduced LPS-induced production of IL-6 and TNF-α [50].

In human athletes, ATX supplementation has been postulated to offer a recovery benefit due to its properties of inhibition of both pro-oxidant and pro-inflammatory intermediates [12]. Unfortunately, this recommendation is based only on in vitro and rodent studies that indicated suppression of TNF-α and IL-1β [27]. A more recent study, however, has not confirmed any apparent effect of a 4-week ATX supplementation on the IL-6, IL-8, IL-10, MCP-1, GCSF, and IL-1ra concentrations in the blood of runners at rest and after a 2.25 h long run at close to 70% maximal oxygen uptake (VO_2max_), where similar exercise-induced increases occurred regardless of the supplementation [51]. The authors claimed that in vitro and rodent findings do not extend to inflammatory-like responses induced by exercise. In their opinion, the immune support from ATX supplementation in the runners was linked to the normalization of post-exercise plasma levels of immune-related proteins including immunoglobulins within 24 h [51].

An exercise-induced inflammatory response has been reported in humans and horses, but the patterns and the occurrence of this reaction are only partially similar. Thus, the effect of ATX described in Nieman et al.’s [51] study cannot be simply extrapolated to horses.

In horses, the exercise-induced changes in cytokine levels in the blood are recognized in two aspects: exercise-induced increases after exertion and the generation of an anti-inflammatory state, which protects the horse from fully developed inflammation after triggering a cytokine response after the effort. Both types of changes seem to be related to the type of exercise. In long-lasting endurance efforts, the post-exercise effect after the longest distances of 120 and 160 km included primarily a two-fold increase in the level of IL-6 accompanied by a 10-fold increase in IL-10 concentration [52]. Thus, it has been postulated that an exercise-induced acute phase response was triggered and promoted by type 1 cytokines, and at the same time, it was strongly inhibited by anti-inflammatory IL-10, preventing the development of inflammation and clinical conditions. At that time, an anti-inflammatory state had already been suggested in racing horses [23] and suspected also in equine endurance athletes. Then, it was shown that in endurance horses, an anti-inflammatory state occurred 3 months after beginning regular training and was expressed by decreases in type 1 cytokines (TNFα and IL-1β), and one month later, the blood level of IL-6 decreased [38].

In racing horses, the different patterns of an anti-inflammatory state triggered by race training have been reported based on cytokine gene expression [23,37]. In 2-year-old Thoroughbreds, training resulted in an overall reduction in inflammatory markers, particularly observed as a decrease in the baseline expression of mRNA for TNFα and a diminishing tendency in the expression of mRNA for IL-1β [23]. The decreases were more pronounced in horses that had received antioxidant supplementation including *Boswellia*, whose anti-inflammatory action is related to the inhibition of nuclear factor-kB [23]. These findings partially correspond with the results obtained in this study, which have indicated that the decreases in the TNFα concentration in blood were also most pronounced but there was no difference related to ATX supplementation, even though both *Boswellia* and ATX act via similar, nuclear factor-kB dependent mechanisms. This inconsistency may be related to the methodology used in the studies, the gene expression level in the study of Horohov et al. [23], and the level of cytokine protein in blood examined in the present study. It is possible that the anti-inflammatory effect of antioxidant supplementation in horses in regular training is slight and can be detected at the stage of gene expression but not during cytokine release, which is influenced by other factors and poses a sum of the production by leukocytes, myocytes, and other cells, as has been shown in treadmill experiments [53,54].

Moreover, Horohov et al. [23] measured only the expression of mRNA for IL-1, IL-6, and TNFα as proinflammatory cytokines and IFNγ as a marker of lymphokine-activated killer (LAK) cells. In a later study on 2-year-old Thoroughbreds, more cytokines and related proteins such as IL-1β, IL-6, IL-8, IL-17A, CCL8, HSP-90, TLR-4, TNFα, TNFSF13B, and VEGFA were examined and an exercise-related anti-inflammatory state was characterized by an inflammation score involving the expression of 10 examined markers and the correlations among them [37]. With 8 weeks of training, the inflammation score either increased or decreased, depending on the training regime. Other studies have reported higher levels of the expression of mRNA for inflammation-related cytokines in trained horses when compared to sedentary animals [34,55]. The present study involved racing Arabians, so the training plan was different than described in the previous studies regarding Thoroughbreds [23,34,37]. Generally, Arabians worked at lower speeds but for longer distances.

Although it is commonly accepted that an intense or long-lasting effort triggers an inflammatory response that does not develop into a clinical condition due to the limiting mechanisms that occur with training, the character of these mechanisms is still not fully understood. Moreover, the patterns of anti-inflammatory mechanisms seem to differ regarding the type of training. The results of the present study indicated that in Arabian racing horses, the anti-inflammatory mechanisms are triggered very early, after 4 weeks of training, and depend mainly on a decrease in TNFα concentration. It has also been shown that after a further 3 months of training the concentration of IFNγ decreases. These decreases seem to be supported by the balance between IL-6 and IL-10, shown as a positive correlation in the present study.

It can be postulated that ATX did not affect the level of cytokines in the blood because their concentrations were very low, indicating the low level of proinflammatory response in general. This is in line with a study on healthy geldings, representing various breeds, supplemented with resveratrol for 3 weeks, which did not change the production of cytokines in stimulated leukocytes [56]. It is also worth mentioning that basic hematological parameters (WBC, RBC, HCT, and HGB) remained within reference intervals and did not show significant variations throughout the study, further suggesting that ATX supplementation did not negatively affect overall health.

The main limitations of this study include the small group of animals that remained in regular training and racing until the end of the observation. The horses were sampled in regular intervals, monthly, so some samples were missed due to the racing plan. Unfortunately, this could not be avoided in a field trial involving performance horses. Another limitation is the fact that this study involved only healthy horses that did not show any abnormalities or poor performance. It cannot be excluded that an anti-inflammatory effect of ATX could have occurred if subclinical inflammation had developed. In such circumstances, some benefits of ATX supplementation may have occurred.

## 5. Conclusions

The results of the present study have shown a notable time-dependent decrease in TNFα and IFNγ concentrations in both groups (ATX and control), indicating a natural inflammatory response modulation toward an anti-inflammatory state due to training. This reduction was evident early, after 4 weeks of training, and persisted, highlighting the effect of exercise itself rather than ATX supplementation. It seems likely that the cytokine response to the training is too slight to be affected by ATX; its anti-inflammatory action deals mainly with chronic and progressive inflammatory conditions.

Positive correlations between TNFα and IFNγ in the early phase of the study and between IL-6 and IL-10 in later stages suggest complex interplays between these cytokines during the inflammatory response and adaptation to training.

While ATX is recognized for its potential antioxidant and anti-inflammatory effects, its supplementation did not significantly alter cytokine levels or enhance performance in Arabian racehorses within the scope of this study. Future research should explore the effects of ATX under different conditions, such as in horses with subclinical inflammation or different training regimens, to fully understand its potential benefits.

## Figures and Tables

**Table 1:** Numbers of races and wins in the astaxanthin (ATX) and control groups during the study period.

	ATX Group (n = 6)	Control Group (n = 8)	*p*-Value
Number of races completed			0.662
0	1	2	
1	0	1	
2	1	1	
3	2	1	
4	0	1	
5	0	0	
6	2	2	
Number of races won			0.852
0	3	4	
1	2	4	
2	1	0	

**Table 2:** Hematological measurements in studied horses in the subsequent months presented as the median and range in the astaxanthin (ATX) and control groups. WBC—white blood cell count, RBC—red blood cell count, HGB—hemoglobin concentration, HCT—hematocrit.

		Month				Friedman Test
	Group	April	May	June	July	*p*-Value
WBC [G/L]	ATX (n = 6)	10.1 (9.7–11.7)	8.9 (7.6–10.2)	9.6 (8.1–12.1)	9.4 (8.3–11.5)	0.126
	Control (n = 8)	9.8 (7.8–10.8)	9.5 (8.1–11.7)	9.9 (8.8–10.9)	9.0 (8.3–10.3)	
RBC [G/L]	ATX (n = 6)	10.3 (9.4–11.8)	9.9 (9.9–11.4)	10.1 (8.9–12.3)	10.4 (9.3–12.1)	0.341
	Control (n = 8)	10.3 (8.1–12.8)	9.9 (4.1–12.8)	10.6 (9.3–13.1)	10.3 (8.7–12.4)	
HGB [g/dL]	ATX (n = 6)	15.0 (14.1–17.4)	14.8 (13.6–17)	14.5 (14.1–15.7)	15.9 (14.0–18.8)	0.509
	Control (n = 8)	15.6 (12.8–17.5)	14.9 (6.1–17.8)	15.7 (15.7–18.9)	15.9 (13.3–18.7)	
HCT [%]	ATX (n = 6)	43.0 (40.5–49.1)	40.7 (37.9–46.4)	42.3 (37.0–55.9)	44.1 (38.1–54.5)	0.362
	Control (n = 8)	43.4 (35.9–49.6)	40.8 (17.4–51.7)	45.1 (37.4–55.7)	43.6 (36.6–51.7)	

**Table 3:** Cytokine and PGE concentrations in the astaxanthin (ATX) and control group, presented as the median and range. TNFα—tumor necrosis factor α, IFNγ—interferon γ, IL-6—interleukin 6, IL-10—interleukin 10, PGE—prostaglandin E.

	Group	Month				Friedman Test
		April	May	June	July	*p*-Value
TNFα	ATX (n = 6)	138 (94.4–372)	6.5 (0–98.9)	1.1 (0–129)	0.6 (0–12.2)	<0.001
	Control (n = 8)	185.4 (0–477)	0 (0–15.6)	7.2 (0–76.7)	0 (0–1.2)	
	*p*-value ^a^	0.846	0.259	0.999	0.256	
IFNγ	ATX (n = 6)	25.9 (17.3–80.0)	19.7 (13.6–43.6)	16.4 (5.5–30.9)	10.8 (7.3–17.3)	0.001
	Control (n = 8)	25.5 (4.6–40.0)	15.5 (10.0–29.1)	8.6 (0–43.6)	10.6 (0–26.4)	
	*p*-value ^a^	0.747	0.299	0.196	0.394	
IL-6	ATX (n = 6)	0 (0–55.0)	0 (0–107.0)	0 (0–99.0)	5.4 (0–108)	0.597
	Control (n = 8)	0 (0–130)	0 (0–0)	0 (0–79.0)	0 (0–10.8)	
	*p*-value ^a^	0.518	0.112	0.928	0.321	
IL-10	ATX (n = 6)	0 (0–0)	0 (0–59.7)	0 (0–13.2)	0.15 (0–0.7)	0.908
	Control (n = 8)	4.0 (0–107)	0 (0–8.5)	0 (0–1.3)	0 (0–7.0)	
	*p*-value ^a^	0.063	0.419	0.323	0.886	
PGE	ATX (n = 6)	2395	2444	2506	2418	0.510
		(2330–2585)	(2389–2521)	(2375–2537)	(2364–2512)	
	Control (n = 8)	2424	2419	2418	2421	
		(1468–2540)	(2324–2490)	(1164–2570)	(2177–2565)	
	*p*-value ^a^	0.949	0.272	0.333	0.948	

^a^ Mann–Whitney U test.

## Data Availability

The original contributions presented in this study are included in the article. Further inquiries can be directed to the corresponding author.

## Abbreviations

ATX, astaxanthin; TNFα, tumor necrosis factor α; IFNγ, interferon γ; IL-6, interleukin 6; IL-10, interleukin 10; PGE: prostaglandin E; HDL, high-density lipoprotein; PSSM, polysaccharide storage myopathy; CK, creatinine kinase; LDH-5, lactate dehydrogenase isoenzyme-5; RBC, red blood cell count; HGB, hemoglobin concentration; HCT, hematocrit; WBC, white blood cell count; NEU, neutrophil count; LYM, lymphocyte count; MON, monocyte count; EOS, eosinophil count; BAS, basophil count; PLT, platelet count; CV, coefficient of variation; IQR, interquartile range; R_s_, Spearman rank correlation coefficient; NF-κB, nuclear factor kappa B; LPS, lipopolysaccharide; LAK, lymphokine-activated killer cells, iNOS, inducible nitric oxide synthase, COX-2, cyclooxygenase 2.
